# Cholecystectomy and Irritable Bowel Syndrome: A Systematic Review and Meta-Analysis of 3,511,681 Patients

**DOI:** 10.3390/jcm15145392

**Published:** 2026-07-09

**Authors:** Jeongin Ra, Shivani Rao, Prakash V. A. K. Ramdass

**Affiliations:** Department of Public Health and Preventive Medicine, School of Medicine, St. George’s University, St. George P.O. Box 7, Grenada; jra@sgu.edu (J.R.); srao3@sgu.edu (S.R.)

**Keywords:** cholecystectomy, gallbladder removal, irritable bowel syndrome, disorder of gut–brain interaction, irritable colon, functional gastrointestinal disorder, IBS, DGBI

## Abstract

**Background/Objectives:** Cholecystectomy, the surgical removal of the gallbladder, is one of the most commonly performed abdominal surgical procedures worldwide. Emerging evidence suggests that cholecystectomy may contribute to the development of irritable bowel syndrome (IBS). However, the association remains incompletely understood. This systematic review and meta-analysis aimed to evaluate the relationship between cholecystectomy and IBS. **Methods:** A systematic search of PubMed, Embase, Scopus, and Web of Science was conducted from database inception to March 2026. Meta-analyses were performed to pool odds ratios (ORs) and prevalence estimates. Heterogeneity, publication bias, and sensitivity analyses were assessed. **Results:** Seventeen studies involving 3,511,681 participants were included in the systematic review. Eight studies were eligible for the meta-analysis of the association between cholecystectomy and IBS, while twelve studies contributed to the prevalence analysis. No statistically significant difference in the odds of IBS was observed between patients who underwent cholecystectomy and controls (OR = 2.46, 95% CI: 0.97–6.22; *p* = 0.056; I^2^ = 91.7%). The pooled prevalence of IBS in post-cholecystectomy patients was 21.0% (95% CI: 9.0–42.0), with high heterogeneity (I^2^ ≈ 99%). Sensitivity analyses did not alter the direction of the pooled estimates following sequential omission of individual studies, and no significant publication bias was detected. **Conclusions:** No statistically significant difference in the pooled odds of IBS was observed between groups, while the pooled prevalence among cholecystectomy patients was approximately 21%, although heterogeneity was substantial. Further research is needed on heterogeneity.

## 1. Introduction

Irritable bowel syndrome (IBS) is a highly prevalent disorder of gut–brain interaction (DGBI), affecting an estimated 13.2% of the population worldwide [1]. Defined as a chronic condition characterized by abdominal pain and altered bowel habits without an identifiable organic cause, IBS manifests through symptoms including diarrhea, constipation, or alternating patterns of both, as well as abdominal pain and bloating [2]. Although IBS rarely leads to severe complications, the economic burden of managing IBS symptoms in the United States alone amounts to 15–30 billion dollars annually, underscoring the profound effect of the disorder on healthcare resources and patient well-being [3].

Despite its high prevalence and considerable healthcare costs, the etiology of IBS remains elusive. Several hypotheses have been proposed to explain its pathophysiology, including potential mechanisms shared with other gastrointestinal conditions that lead to disturbance of the gut–brain axis [4]. Factors such as biliary disease or biliary surgery are proposed to elevate IBS risk, suggesting a complex interplay between these organs and the sensory processing pathways of the gut [5]. However, while the shared mechanisms of altered sensory processing have been previously investigated, the relationship between IBS and other gastrointestinal disorders remains inadequately defined. Particularly, the association between biliary disease and IBS is not fully understood.

Cholecystectomy, one of the most commonly performed gastrointestinal surgical procedures worldwide, involves the removal of the gallbladder and has been increasingly investigated for its potential role in the development of functional gastrointestinal disorders [6]. Several biological mechanisms have been proposed to explain a potential association between cholecystectomy and IBS. Following gallbladder removal, continuous bile flow into the intestine may alter bile acid metabolism, intestinal transit, and gut microbial composition, potentially contributing to the development of IBS symptoms [7]. In addition, alterations in the gut microbiota [8], increased intestinal permeability [9], low-grade mucosal inflammation [10], visceral hypersensitivity [11], and dysregulation of the gut–brain axis [12] following cholecystectomy may further contribute to the development or exacerbation of IBS symptoms. These mechanisms suggest that cholecystectomy could influence gastrointestinal function beyond the biliary system and potentially predispose susceptible individuals to functional bowel disorders.

Nevertheless, research on the relationship between cholecystectomy and IBS has yielded mixed results. Some studies suggest that cholecystectomy may increase the risk of developing IBS [13], while others indicate it could have a protective effect [14]. Given these conflicting findings, this systematic review and meta-analysis aimed to investigate and quantify the relationship between cholecystectomy and IBS, providing a clearer understanding of their potential interplay. These findings may help inform future clinical research and patient management.

## 2. Materials and Methods

### 2.1. Study Protocol and Registration

This research was conducted following the guidelines established by the Preferred Reporting Items for Systematic Reviews and Meta-Analyses (PRISMA) and the PRISMA-Protocol statement [15], with registration in the PROSPERO database (University of York, United Kingdom) https://www.crd.york.ac.uk/PROSPERO/view/CRD420261401418 (accessed on 22 May 2026). To guide the literature search and article selection, the research question was developed using the PICO (Population, Intervention, Comparison, Outcome) framework [16]. The PRISMA Checklist is shown in Supplementary Materials.

### 2.2. Review Question According to the PICO Framework

The review question of this study, according to the PICO framework, was as follows:

1. Population: Human adults, age ≥ 18 years
2. Intervention: Patients who have had a cholecystectomy
3. Comparator: Patients without cholecystectomy
4. Outcome: Irritable bowel syndrome

### 2.3. Data Sources and Searches

The search for relevant literature was conducted using four databases: PubMed, Embase, Scopus, and Web of Science. Search strategies for each database were as follows: for PubMed, the search strategy was (“Cholecystectomy”[Mesh] OR “gallbladder removal”[tiab]) AND (“Irritable Bowel Syndrome”[Mesh] OR “disorder of gut–brain interaction”[tiab]); for Embase, the search strategy was (‘cholecystectomy’/exp OR ‘gallbladder removal’, ti) AND (‘irritable colon’/exp OR ‘disorder of gut–brain interaction’, ti); for Scopus, the search strategy was TITLE-ABS-KEY((“cholecystectomy” OR “gallbladder removal”) AND (“irritable bowel syndrome” OR “disorder of gut–brain interaction”)); for Web of Science, the search strategy was TS = ((“cholecystectomy” OR “gallbladder removal”) AND (“irritable bowel syndrome” OR “disorder of gut–brain interaction”)).

The database search was from inception to March 2026, with a restriction to English-language publications. Citation files obtained from the database searches were imported into Zotero software, version 9.0.5 (Corporation for Digital Scholarship, Vienna, VA, USA), and duplicates were removed.

### 2.4. Study Selection and Eligibility

Initially, two reviewers (J.R. and S.R.) screened the titles and abstracts to assess eligibility based on predefined inclusion criteria, which encompassed cross-sectional, cohort, case–control, and Mendelian randomization (MR) studies investigating the association of cholecystectomy and IBS, or studies with data on the prevalence of IBS in patients who had a cholecystectomy. Exclusion criteria were case reports, conference papers, opinions, editorials, abstracts, animal studies, and studies without full-text availability. The second screening phase (done by J.R., S.R., and P.V.A.K.R.) involved a thorough review of the full manuscripts, further refining the selection based on the inclusion criteria and relevance to the research question. Articles that met the criteria with available numerical data were deemed suitable for the meta-analysis. All other articles meeting the eligibility criteria were included in the systematic review. In cases of discrepancies regarding article eligibility, disagreements were resolved through discussion.

### 2.5. Data Extraction

Data was extracted and inserted into an Excel file with the following headings: study (last name of first author, year of publication), country, study design, criteria used to diagnose IBS, confounders assessed in the study, sample size, prevalence of IBS, number of patients with IBS in those with and without cholecystectomy (controls), number of patients without IBS in those with/without cholecystectomy, median follow-up time (in years) after cholecystectomy, and IBS subtypes. For studies reporting raw event data, crude (unadjusted) odds ratios were calculated directly from the reported 2 × 2 contingency tables. When raw data were unavailable, the published effect estimate and corresponding 95% confidence interval were extracted from the original study. Because adjusted effect estimates were not consistently reported across the included studies, crude effect estimates were preferentially used to ensure methodological consistency and comparability across studies.

### 2.6. Data Synthesis and Analysis

All statistical analyses were performed using RStudio software, version 4.4.3 (R Foundation for Statistical Computing, Vienna, Austria), primarily utilizing the “meta” and “metafor” packages. Random-effects meta-analyses were conducted to pool odds ratios (ORs) and corresponding 95% confidence intervals (CIs) for the association between IBS and cholecystectomy, with heterogeneity assessed using Cochran’s Q statistic, τ^2^, and the I^2^ statistic. Forest plots were generated to visually summarize pooled effect estimates and subgroup analyses according to study design, country, and IBS diagnostic criteria. Subgroup analyses according to study design were performed to evaluate differences between observational studies. Because MR studies investigate genetically inferred causal relationships rather than conventional observational associations, these studies were excluded from the overall pooled analysis and were included in the systematic review only.

Leave-one-out sensitivity analyses were performed using influence diagnostics to evaluate the robustness of the pooled estimates after sequential omission of individual studies. Baujat plots were generated to identify studies contributing most substantially to heterogeneity and overall effect size influence. Additional influence diagnostics included assessment of studentized residuals, Cook’s distance, DFFITS, and covariance ratios. Publication bias and small-study effects were evaluated through visual inspection of funnel plots, alongside formal statistical testing using Egger’s regression test [17] and Begg’s rank correlation test [18]. Meta-regression analyses were performed to investigate potential sources of between-study heterogeneity, including country, study design, sample size, IBS diagnostic criteria, Newcastle–Ottawa Scale (NOS) [19] quality rating, and follow-up duration. Statistical significance was defined as a two-tailed *p*-value < 0.05.

### 2.7. Risk of Bias (Quality) Assessment

Three reviewers (J.R., S.R., and P.V.A.K.R.) assessed the risk of bias (ROB) of cohort studies with the NOS [19], cross-sectional studies with an adapted version of the NOS [20]. The NOS is a quality-assessment tool for observational studies in meta-analyses and systematic reviews based on study group selection, comparability, and determination of exposure and outcome. The overall scores were recorded, and any discrepancy was resolved through discussion. Cohort studies were classified as low risk (8–9 stars), moderate risk (6–7 stars), or high risk (≤5 stars). Cross-sectional studies were classified as low risk (7–10 points), moderate risk (4–6 points), or high risk (≤3 points). Because no universally accepted risk-of-bias tool exists specifically for Mendelian randomization studies, methodological quality was assessed using the Quality of Genetic Association Studies (Q-Genie) instrument [21], which was originally developed for genetic association studies and adapted for the present review. The Q-Genie assesses methodological quality across seven key domains: (1) gene/instrument selection and relevance, (2) control of confounding, (3) selection bias, (4) measurement bias, (5) statistical analysis, (6) replication and validation of findings, and (7) interpretation of results. Each domain was assessed independently, and studies were classified according to their overall methodological quality. Consistent with the adapted scoring system used in this review, studies scoring 6–7 points were considered at low risk of bias, 4–5 points at moderate risk of bias, and ≤3 points at high risk of bias.

## 3. Results

### 3.1. Study Selection

**Figure 1:**
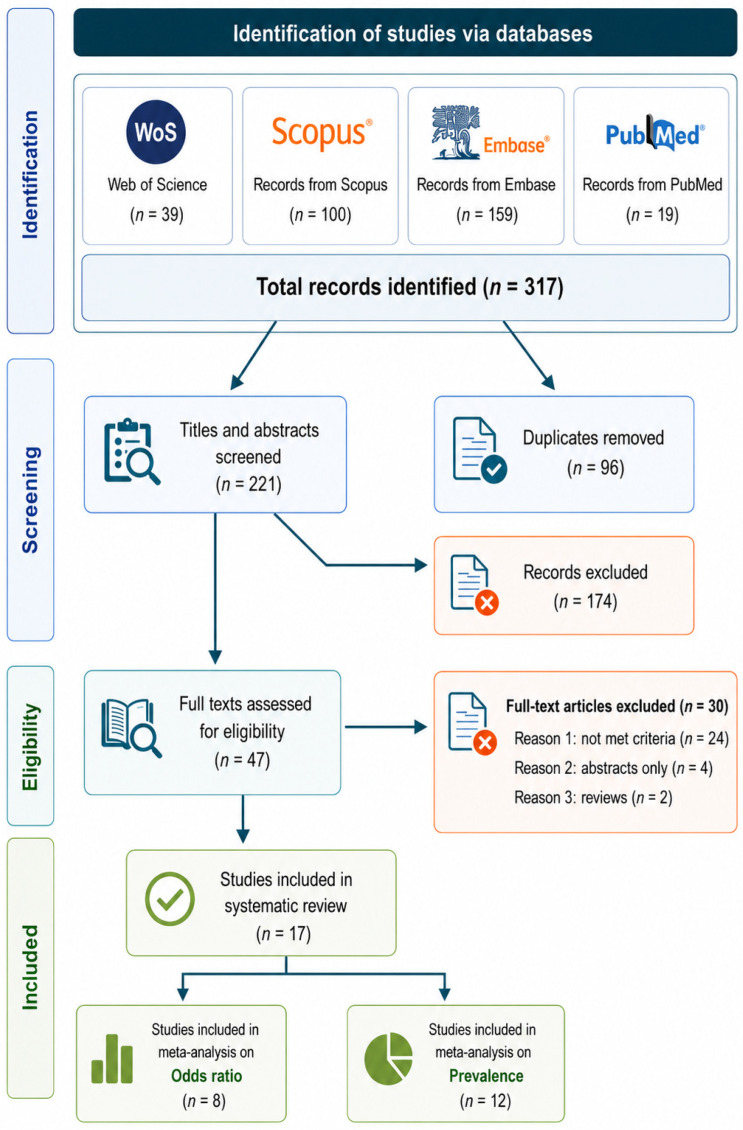
Modified PRISMA flow diagram for study selection.

Figure 1 illustrates the PRISMA flow diagram for study selection. A total of 317 records were initially identified through database searching, including records from Web of Science (*n* = 39), Scopus (*n* = 100), Embase (*n* = 159), and PubMed (*n* = 19). After the removal of 96 duplicate records, 221 titles and abstracts were screened, of which 174 records were excluded based on irrelevance to the study objectives. Subsequently, 47 full-text articles were assessed for eligibility. Of these, 30 full-text articles were excluded, and ultimately 17 studies [5,7,13,14,22,23,24,25,26,27,28,29,30,31,32,33,34] were included in the systematic review. Among these, 8 studies were included in the meta-analysis evaluating the odds ratio between IBS and cholecystectomy, while 12 studies were included in the meta-analysis on the prevalence of IBS in patients who had cholecystectomy.

### 3.2. Characteristics of Included Studies

The studies included in this review were conducted across at least 11 countries, with the majority originating from the United Kingdom (4 studies), China (4 studies), Mexico (2 studies), one study each from 5 different countries, and one multinational study conducted across 26 countries. The studies were published between 1993 and 2025. Among the included studies, the predominant study design was prospective cohort (10 studies), followed by cross-sectional studies (4 studies) and MR studies (3 studies). Although ten studies employed prospective designs, only two (Heaton et al. and Mjåland et al.) explicitly assessed and excluded pre-existing IBS prior to cholecystectomy using validated criteria [22,23]. The MR studies utilized data from the Genome-Wide Association Studies (GWAS) and the Dutch Project. Most studies utilized the Rome I (1) [35], Rome II (3) [36], Rome III (6) [37] and Rome IV (2) [38] criteria for the diagnosis of IBS, whereas 3 studies employed the Manning criteria [39] and 2 studies did not clearly specify the diagnostic criteria. Several studies adjusted for important confounding variables, including age, sex, body mass index (BMI), sociodemographic factors, psychiatric history, comorbidities, medications, dietary factors, gastroesophageal reflux disease (GERD), coping strategies, and health behaviors. Follow-up duration after cholecystectomy was reported in ten studies and ranged from 0.5 to 12.7 years, although several studies did not specify follow-up time. IBS subtype analyses were reported in six studies and included constipation-predominant IBS (IBS-C), diarrhea-predominant IBS (IBS-D), mixed IBS (IBS-M), and unclassified IBS (IBS-U), with IBS-D being the most frequently evaluated subtype. The MR studies additionally accounted for genetic variants and population stratification through GWAS methodologies. Detailed study characteristics, including country, study design, IBS diagnostic criteria, confounders and sample size, are summarized in Table 1.

### 3.3. Systematic Review

**Figure 2:**
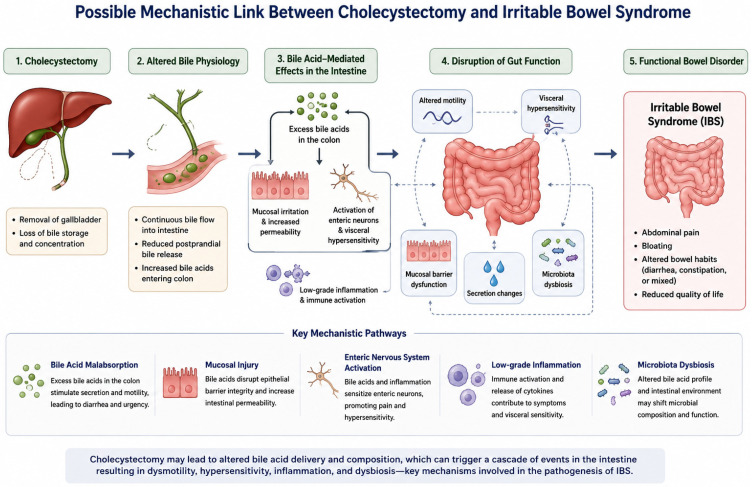
Proposed mechanistic pathways between cholecystectomy and IBS.

The 17 studies included in the systematic review demonstrated considerable variability in study design, sample size, population characteristics, and diagnostic criteria for IBS and cholecystectomy-related outcomes. Overall, most observational and cohort studies suggested a positive association between cholecystectomy and subsequent IBS, particularly diarrhea-predominant IBS. Early population-based studies by Heaton et al. [22] and Hearing et al. [34] reported increased bowel urgency and altered bowel habits following cholecystectomy, although severe diarrhea was uncommon. Prospective cohort studies further demonstrated that patients with pre-existing IBS symptoms or functional gastrointestinal disorders experienced poorer postoperative outcomes and persistent abdominal symptoms after surgery. More recent studies from Mexico, Korea, China, and the United Kingdom reported significantly increased risks of IBS, particularly IBS with diarrhea, following cholecystectomy. Cross-sectional and registry-based studies also demonstrated higher frequencies of abdominal pain, dyspepsia, bloating, gut dysbiosis, and healthcare utilization among post-cholecystectomy patients. MR studies consistently supported a potential causal relationship between cholecystectomy and IBS risk, with reported odds ratios ranging from approximately 3.0 to 7.6 [7,13,31]. However, not all studies demonstrated a definitive association, as some prospective cohorts found no statistically significant increase in IBS incidence after laparoscopic cholecystectomy. The narrative synthesis suggests that cholecystectomy may contribute to the development of IBS through alterations in bile acid metabolism, accelerated intestinal transit, gut microbiota dysbiosis, visceral hypersensitivity, and persistent functional gastrointestinal disturbances, with the proposed mechanistic pathways depicted in Figure 2.

### 3.4. Odds of IBS in Cholecystectomy Patients

**Figure 3:**
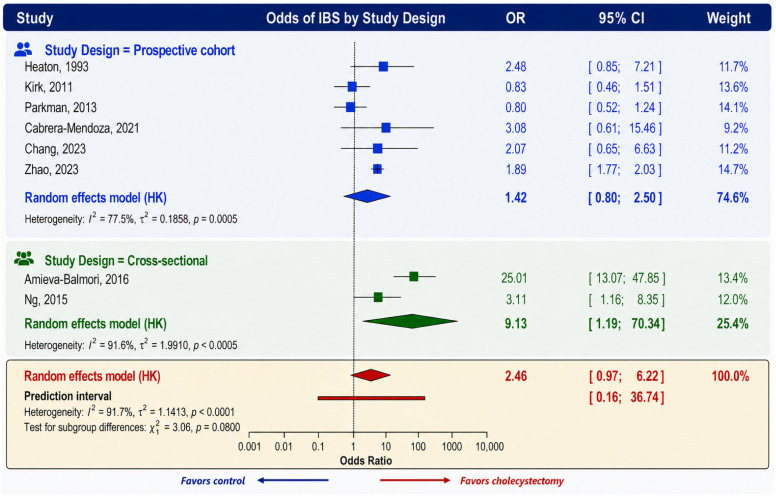
Forest plot showing meta-analysis of the odds of IBS in patients who had cholecystectomy [5,14,22,25,27,28,30,32].

No statistically significant difference in the pooled odds of IBS across the eight studies was observed between patients who underwent cholecystectomy and controls (OR = 2.46, 95% CI: 0.97–6.22; I^2^ ≈ 99%; *p* = 0.056). The prediction interval was wide (0.16–36.74), indicating substantial between-study variability. Heterogeneity was high (I^2^ = 91.7%, *p* < 0.001). In subgroup analysis, prospective cohort studies showed a weaker and non-significant association (OR = 1.42, 95% CI: 0.80–2.50), while cross-sectional studies showed a higher but imprecise estimate (OR = 9.13, 95% CI: 1.19–70.34). The difference between study designs was not statistically significant (*p* = 0.080). The results are shown in Figure 3.

### 3.5. Prevalence of IBS in Cholecystectomy Patients by IBS Criteria

**Figure 4:**
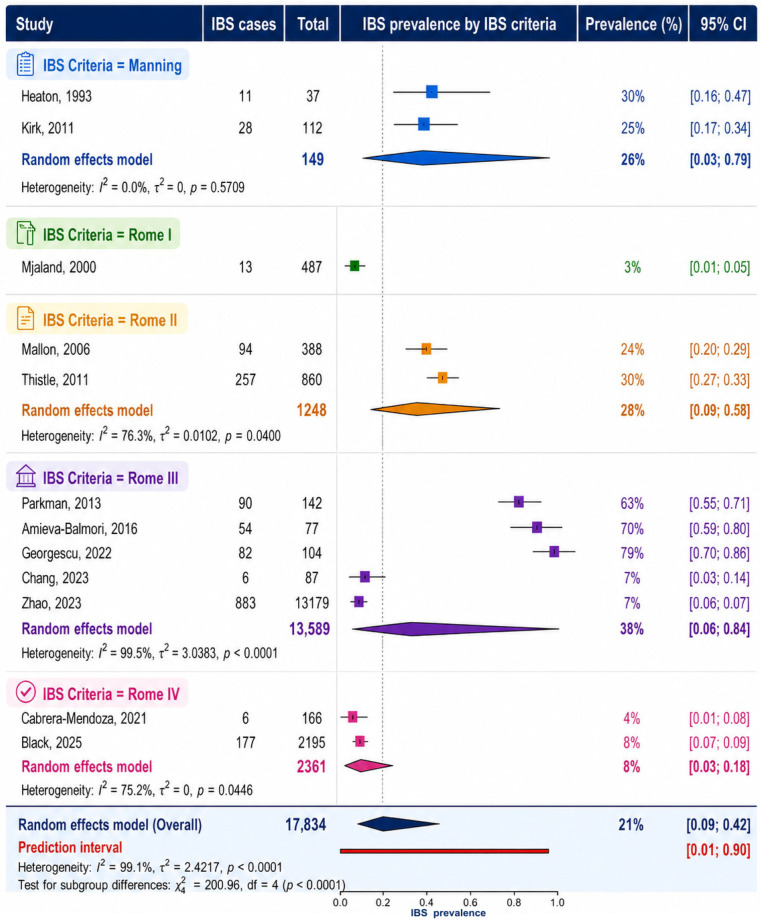
Forest plot showing meta-analysis of prevalence of IBS in patients who had cholecystectomy by IBS criteria [5,14,22,23,24,25,26,27,28,29,30,32,33].

Figure 4 presents the subgroup analysis of IBS prevalence in patients who had cholecystectomy according to the IBS diagnostic criteria used. Studies utilizing Rome III criteria reported the highest pooled prevalence (37.7%, 95% CI: 6.4–84.4%), followed by Rome II (27.5%, 95% CI: 9.4–58.1%) and Manning criteria (26.2%, 95% CI: 3.2–79.1%). In contrast, studies using Rome IV criteria demonstrated a markedly lower prevalence (7.8%, 95% CI: 3.1–18.3%), while the single Rome I study reported a prevalence of 2.7% (95% CI: 1.6–4.5%). Heterogeneity was minimal among studies using Manning criteria (I^2^ = 0%) but remained substantial for Rome II (I^2^ = 76.3%), Rome III (I^2^ = 99.5%), and Rome IV (I^2^ = 75.2%) criteria.

### 3.6. Prevalence of IBS in Cholecystectomy Patients by Study Design

**Figure 5:**
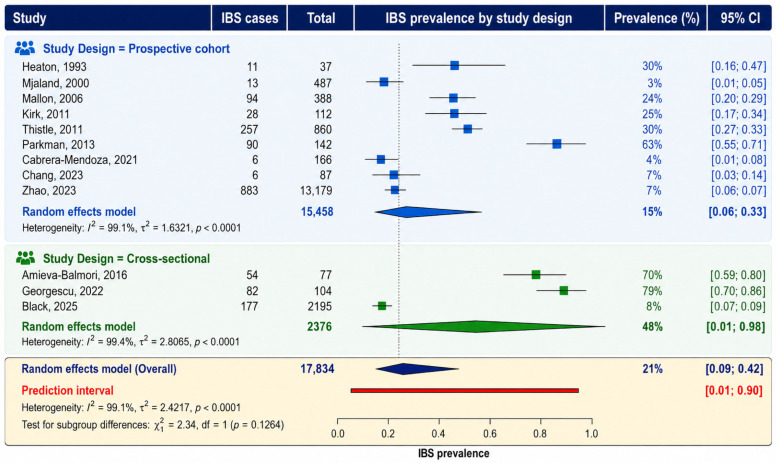
Forest plot showing meta-analysis of the prevalence of IBS in patients who had cholecystectomy by study design [5,14,22,23,24,25,26,27,28,29,30,32,33].

Figure 5 presents the subgroup meta-analysis examining the prevalence of IBS in patients who had cholecystectomy by study design. When stratified by study design, cross-sectional studies reported a higher pooled prevalence of IBS after cholecystectomy than prospective cohort studies (47.6%, 95% CI: 1.4–98.4% vs. 15.1%, 95% CI: 6.1–32.7%). However, the difference between study designs did not reach statistical significance (*p* = 0.1264). Both subgroups exhibited considerable heterogeneity, with I^2^ values of 99.4% for cross-sectional studies and 99.1% for prospective cohort studies, indicating substantial variability among the included studies irrespective of study design.

### 3.7. Prevalence of IBS in Cholecystectomy Patients by Study Country

**Figure 6:**
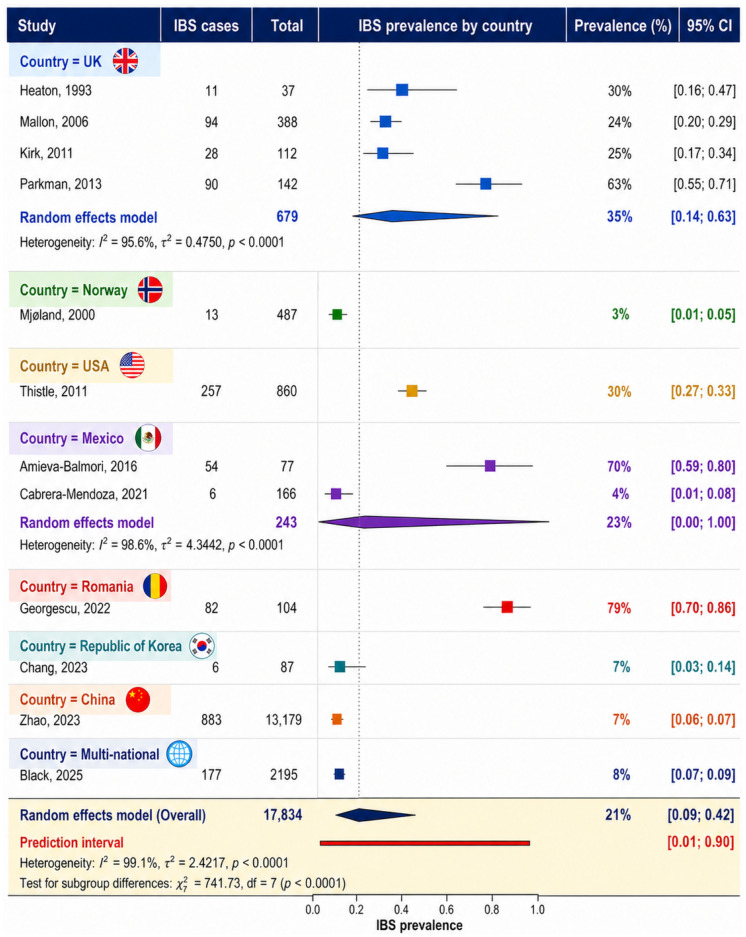
Forest plot showing meta-analysis of the prevalence of IBS in patients who had cholecystectomy by study country [5,14,22,23,24,25,26,28,29,30,32,33].

As shown in Figure 6, the subgroup analysis by country demonstrated significant variation in the prevalence of IBS among patients who underwent cholecystectomy (*p* <0.001). The highest pooled prevalence was observed in Romania (78.8%, 95% CI: 69.9–85.7%), followed by the United Kingdom (34.6%, 95% CI: 14.3–62.7%), the United States (29.9%, 95% CI: 26.9–33.0%), and Mexico (22.8%, 95% CI: 0.0–100.0%). Lower prevalence estimates were reported in the Republic of Korea (6.9%, 95% CI: 3.1–14.5%), China (6.7%, 95% CI: 6.3–7.1%), Norway (2.7%, 95% CI: 1.6–4.5%), and the multinational (participants from 26 countries) study (8.1%, 95% CI: 7.0–9.3%). Substantial heterogeneity was present within the UK (I^2^ = 95.6%) and Mexican (I^2^ = 98.6%) subgroups.

**Figure 7:**
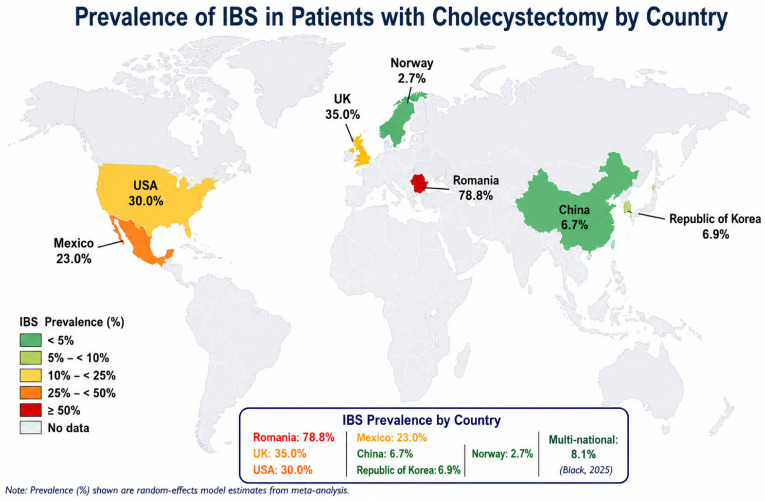
Pooled prevalence estimates of IBS following cholecystectomy by geographic region [33].

Figure 7 provides a geographic visualization of the pooled prevalence estimates of IBS following cholecystectomy across the countries represented in the meta-analysis. Consistent with the subgroup analysis, Romania exhibited the highest prevalence, while Norway, China, and the Republic of Korea demonstrated the lowest prevalence estimates. Countries are color-coded according to prevalence categories, illustrating a marked geographic variation in IBS occurrence after cholecystectomy. The map further highlights the concentration of higher prevalence estimates in parts of Europe and North America compared with East Asia, although these findings should be interpreted cautiously given the limited number of studies available from several regions and the substantial heterogeneity observed within some country-specific subgroups.

### 3.8. Prevalence of IBS in Cholecystectomy Patients by NOS Quality Rating

**Figure 8:**
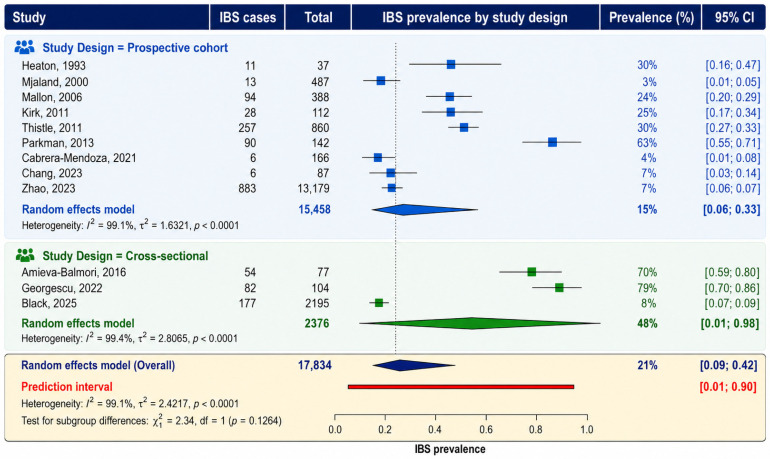
Forest plot showing meta-analysis of the prevalence of IBS in patients who had cholecystectomy by NOS quality rating [5,14,22,23,24,25,26,28,29,30,32].

Subgroup analysis according to NOS quality rating demonstrated no significant differences in pooled IBS prevalence following cholecystectomy between study quality categories (*p* = 0.128). Studies classified as low risk of bias (*n* = 4) yielded a pooled prevalence of 12% (95% CI: 2–45%), whereas studies classified as moderate risk of bias (*n* = 7) demonstrated a higher pooled prevalence of 32% (95% CI: 9–69%). Substantial heterogeneity was observed within both the low-risk (I^2^ = 97.1%) and moderate-risk (I^2^ = 99.3%) subgroups. Overall, the pooled prevalence of IBS after cholecystectomy was 23% (95% CI: 9–47%), with a wide prediction interval of 1–92%, indicating considerable variability in prevalence estimates across studies. These findings suggest that study quality, as assessed by the NOS, did not significantly influence the overall prevalence estimates, suggesting that study quality did not influence the pooled prevalence estimates. These findings are shown in Figure 8.

### 3.9. Influence Diagnostics

**Figure 9:**
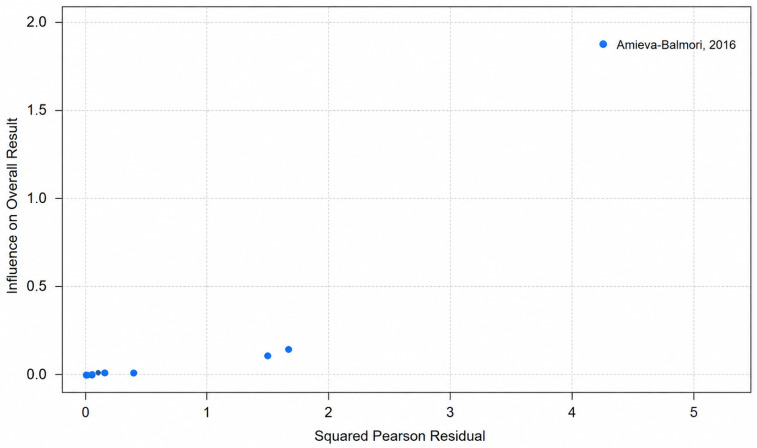
Baujat plot of study influence. Each blue dot represents a study [28].

Influence diagnostics demonstrated that most studies had minimal influence on the overall pooled effect estimate, with low studentized residuals, Cook’s distances, DFFITS values, and covariance ratios clustering near the reference thresholds. The Baujat plot, shown in Figure 9, identified the study by Amieva-Balmori et al. [28] as the most influential study, contributing substantially to both heterogeneity and the overall pooled effect size.

### 3.10. Sensitivity Analysis

**Figure 10:**
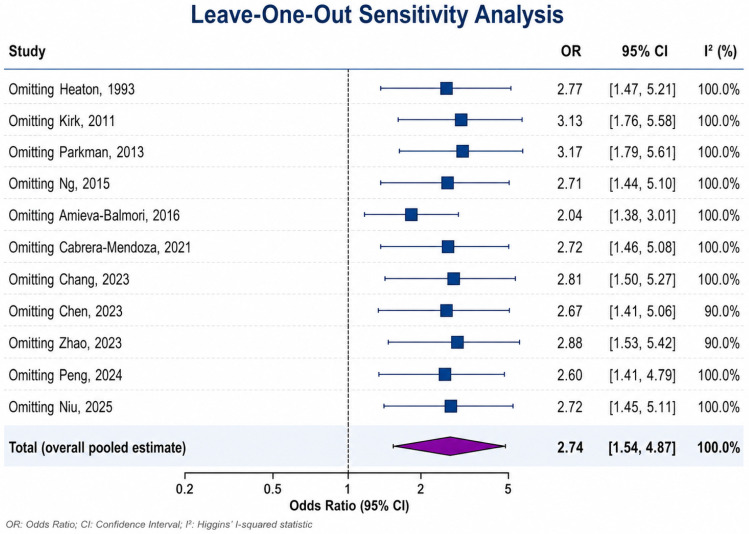
Leave-one-out sensitivity analysis [5,7,13,14,22,25,27,28,30,31,32].

The leave-one-out sensitivity analysis in Figure 10 shows that exclusion of individual studies, including that by Amieva-Balmori et al. [28], did not materially alter the direction or statistical significance of the pooled association, indicating that the overall findings were robust. Although heterogeneity remained high across analyses, no single study alone accounted for the observed between-study variability.

### 3.11. Meta-Regression

Among the evaluated moderators, only study design significantly influenced the pooled prevalence estimates (QM = 7.66, *p* = 0.006), explaining approximately 41.0% of the between-study heterogeneity (R^2^ = 40.96%). Studies with a prospective cohort design reported significantly lower IBS prevalence than cross-sectional studies (β = −2.79, 95% CI: −4.76 to −0.81). Despite this, substantial residual heterogeneity remained (I^2^ = 99.0%; QE = 881.48, *p* < 0.0001). No significant associations were observed for country (QM = 6.84, *p* = 0.336; R^2^ = 8.0%), sample size (QM = 1.99, *p* = 0.159; R^2^ = 9.4%), IBS diagnostic criteria (QM = 4.97, *p* = 0.290; R^2^ = 9.0%), ROB quality rating (QM = 1.61, *p* = 0.204; R^2^ = 5.8%), or follow-up duration (QM = 0.005, *p* = 0.9457; R^2^ = 0.0%), indicating that these variables did not significantly explain the observed heterogeneity. A multivariable meta-regression including study design, sample size, NOS rating, and follow-up duration demonstrated a borderline overall effect (QM = 8.79, *p* = 0.067), with study design remaining the only independent significant predictor (β = −2.97, *p* = 0.041). Collectively, these findings suggest that differences in study design account for a meaningful proportion of the heterogeneity, whereas the remaining variability is likely attributable to other unmeasured methodological or clinical factors.

Despite extensive subgroup analyses and meta-regression, the substantial heterogeneity observed (I^2^ = 96.9% in the main association analysis and I^2^ up to 99.1% in prevalence analyses) remained largely unexplained. Notably, several potential sources of heterogeneity could not be fully evaluated due to inconsistent reporting across studies. These include differences in the timing of IBS assessment relative to surgery, incomplete reporting of IBS subtypes, and variability in the definition of cholecystectomy (e.g., laparoscopic vs. open approach and emergency vs. elective procedures). Additionally, the use of different IBS diagnostic criteria (Manning, Rome I–IV) appeared to influence prevalence estimates, with Rome IV criteria yielding notably lower estimates than earlier versions. Geographic variation was also substantial, ranging from 2.7% in Norway to 78.8% in Romania, potentially reflecting differences in healthcare systems, dietary patterns, genetic susceptibility, or diagnostic practices. These factors collectively underscore the complexity of synthesizing evidence across heterogeneous study populations and highlight the need for standardized methodology in future research.

### 3.12. Publication Bias

**Figure 11:**
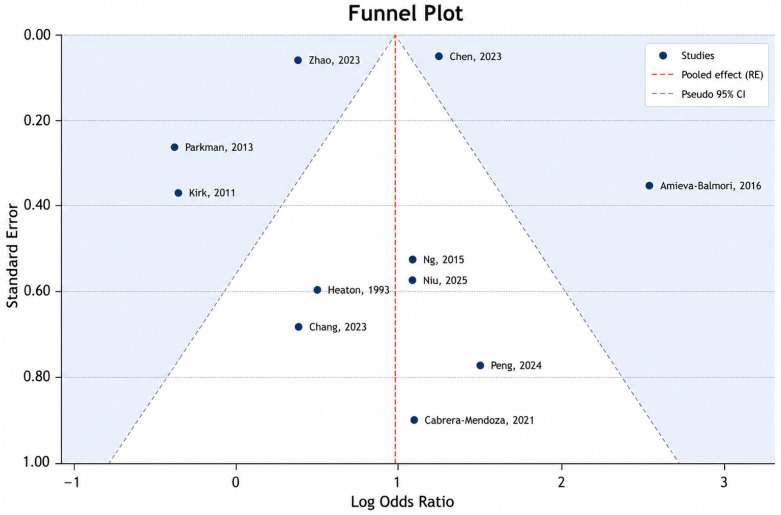
Funnel plot for publication bias [5,7,13,14,22,25,27,28,30,31,32].

Inspection of the funnel plot, shown in Figure 11, demonstrated some visual asymmetry, primarily due to a small number of studies with relatively large effect estimates, including the study by Amieva-Balmori et al. [28]. Nevertheless, formal assessments for publication bias were not statistically significant. Egger’s regression test showed no evidence of funnel plot asymmetry (z = 0.53, *p* = 0.597), and Begg’s rank correlation test was likewise non-significant (Kendall’s τ = 0.13, *p* = 0.648). However, given the limited number of studies (*n* = 11) and high heterogeneity, these tests may lack sufficient statistical power to meaningfully assess publication bias, and thus should be interpreted cautiously. Nevertheless, these findings suggest that although substantial heterogeneity was present, there was no statistically significant evidence of small-study effects or publication bias.

### 3.13. Risk of Bias Assessment

**Figure 12:**
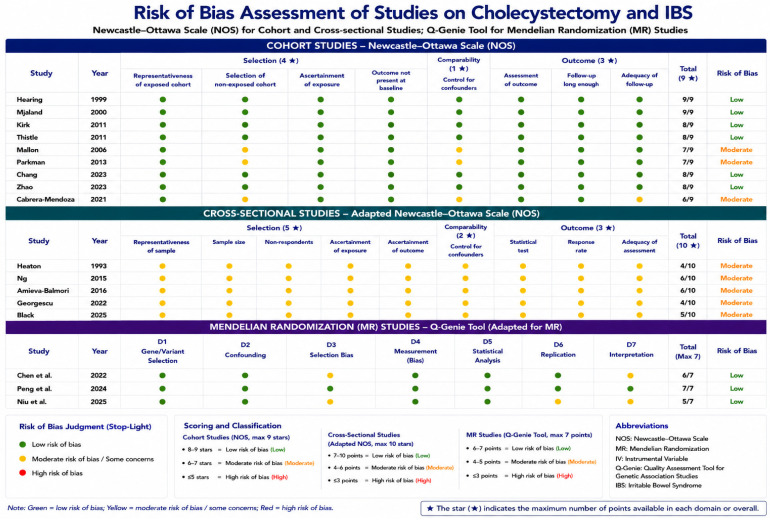
Risk of bias assessment [5,7,13,14,22,23,24,25,26,27,28,29,30,31,32,33,34].

The risk-of-bias assessment is presented in Figure 12. Overall, the methodological quality of the included studies ranged from low to moderate risk of bias, with no studies classified as high risk. Among the cohort studies evaluated using the NOS, five studies were classified as low risk of bias (scores 8–9/9), while four studies were classified as moderate risk of bias (scores 6–7/9).

All cross-sectional studies were classified as moderate risk of bias according to the adapted NOS criteria (scores 4–6/10). Common limitations included concerns regarding sample representativeness, non-response bias, control for confounding variables, and adequacy of outcome assessment. Heaton et al. (1993) [22] and Georgescu et al. (2022) [29] received the lowest scores (4/10), whereas Ng et al. (2015) [27] and Amieva-Balmori et al. (2016) [28] achieved the highest cross-sectional scores (6/10).

The three MR studies assessed using the adapted Q-Genie tool were all classified as having low risk of bias, with overall scores ranging from 5 to 7 out of 7 points. All studies demonstrated appropriate genetic instrument selection, adequate control for confounding, robust statistical analyses, and satisfactory interpretation of findings.

Collectively, these findings indicate that the overall body of evidence was of acceptable methodological quality, although residual confounding and substantial heterogeneity among observational studies should be considered when interpreting the pooled estimates.

## 4. Discussion

Our systematic review and meta-analysis encompasses data from more than 3.5 million participants across 17 studies. It provides a comprehensive synthesis of the available evidence suggesting a possible association between cholecystectomy and IBS, although the pooled association did not reach statistical significance and should be interpreted cautiously given the substantial between-study heterogeneity. The pooled analysis suggested that individuals who underwent cholecystectomy may have higher odds of developing IBS than controls (OR = 2.46); however, the association did not reach statistical significance (95% CI: 0.97–6.22, *p* = 0.056, I^2^ = 91.7%). Sensitivity analyses demonstrated that the direction of the association remained generally consistent after sequential omission of individual studies, although substantial heterogeneity persisted. The observational findings were also broadly consistent with Mendelian randomization studies [7,13,31], which independently suggested a potential causal relationship; however, these results should be interpreted as complementary rather than confirmatory because MR studies evaluate genetically inferred causal effects using a different methodological framework. In addition, the pooled prevalence analysis estimated that approximately one in five patients experienced IBS following cholecystectomy; however, this estimate should be interpreted cautiously because between-study heterogeneity was extremely high (I^2^ ≈ 99%) and the prediction interval was wide. These findings suggest that some patients may experience persistent gastrointestinal symptoms following gallbladder removal, although the true prevalence is likely to vary substantially across different populations and clinical settings. Collectively, these findings are consistent with the hypothesis that cholecystectomy may contribute to persistent functional gastrointestinal symptoms in susceptible individuals, although the current evidence remains uncertain because of the substantial residual heterogeneity observed across studies. This potential association may occur through alterations in bile acid synthesis and metabolism [40], gut microbiota composition [41], intestinal motility [42], and gut–brain axis regulation [12].

The substantial heterogeneity observed in this meta-analysis has important implications for the interpretation and clinical application of our findings. Although the pooled odds ratio (OR = 2.74) suggests that cholecystectomy is associated with a nearly threefold increase in the odds of IBS, this summary estimate should be interpreted with caution, given the wide prediction interval (0.16–36.74 in the association analysis; 1–92% in the prevalence analysis). The prediction interval indicates that the true effect in a future individual study could range from a protective effect to a very large harmful effect, reflecting the considerable uncertainty in the evidence base. This wide interval limits the precision with which we can estimate the magnitude of risk in any given patient population or clinical setting. Furthermore, the substantial geographic variability suggests that the risk of developing IBS after cholecystectomy may differ markedly between populations, potentially due to differences in genetic susceptibility, dietary patterns, gut microbiota composition, or healthcare systems. Therefore, our findings are best viewed as suggestive of a possible association that warrants further investigation rather than as a precise or universally generalizable estimate of risk. Clinicians should consider the possibility of post-cholecystectomy IBS but should also recognize that the likelihood of developing clinically significant symptoms may vary considerably between individual patients and populations.

While IBS affects approximately 3.8–13.2% of the general population worldwide [1,43], our meta-analysis estimated a pooled IBS prevalence of approximately 20% among patients who had undergone cholecystectomy. However, this estimate should be interpreted cautiously because heterogeneity between studies was extremely high (I^2^ ≈ 99%), indicating considerable variation across study populations. These findings suggest that IBS may occur more frequently in some post-cholecystectomy populations, although the magnitude of this association likely varies considerably according to patient characteristics, study design, and diagnostic criteria. Notably, given that cholecystectomy is one of the most commonly performed abdominal surgical procedures worldwide [6], even a modest increase in the risk of IBS could translate into a substantial clinical and public health burden, affecting a large number of patients annually. Interestingly, both cholecystectomy and IBS are more frequently observed in women [43,44]. These findings are clinically important given the substantial burden of IBS on quality of life [45], healthcare utilization [46], and psychological well-being [47].

Cholecystectomy has also been associated with persistent functional gastrointestinal symptoms, including abdominal pain, bloating, dyspepsia, and altered bowel habits, even in the absence of structural abnormalities [30]. Furthermore, psychological factors and pre-existing disorders of gut–brain interaction may predispose certain individuals to persistent postoperative gastrointestinal symptoms, potentially amplifying symptom perception and chronicity [48]. Collectively, these findings suggest that cholecystectomy may act as a physiological and functional trigger for IBS symptoms in susceptible patients through gut–brain axis dysregulation [49].

Several mechanisms may explain the higher prevalence of IBS symptoms following cholecystectomy. One of the most prominent mechanisms is the disruption of bile acid metabolism [41]. The gallbladder plays a crucial role in storing and releasing bile salts, which aid in fat digestion and help regulate gut motility [50]. Following gallbladder removal, bile is continuously released into the small intestine, regardless of food intake, leading to increased bile acid concentration in the small intestine [51]. Excessive bile acids reaching the colon can irritate the intestinal mucosa, leading to diarrhea, a common IBS symptom [52]. The continuous release of bile acids can also alter colonic motility, contributing to diarrhea-predominant IBS (IBS-D) or triggering colonic spasms, which may result in symptoms of IBS with constipation (IBS-C) [53]. High bile acid concentrations have been shown to sensitize enteric nerves, amplifying visceral pain and discomfort, which is characteristic of IBS [54]. Three studies [5,25,32] observed that IBS-D (characterized by more frequent and looser stools) was more prevalent among patients who underwent cholecystectomy. Notably, a study by Peleman et al. further demonstrated that patients with IBS-D had higher levels of secretory bile acids in their stools and reduced intestinal transit time, regardless of whether they had bile acid malabsorption (BAM) [55].

Another possible mechanism explaining the increased likelihood of IBS in patients who had cholecystectomy is gut microbiota alterations (dysbiosis) [8]. Without the gallbladder, the continuous flow of bile into the small intestine can disrupt the microbial environment, leading to small intestinal bacterial overgrowth (SIBO), which is associated with both IBS and post-cholecystectomy syndrome [56]. A meta-analysis conducted by Zhuang et al. demonstrated that gut microbiota dysbiosis, particularly involving bacteria such as *Bifidobacteria*, *Lactobacillus*, *Escherichia coli*, and *Enterobacter*, significantly influences the pathogenesis of IBS [57].

However, reverse causation cannot be excluded. Patients with undiagnosed IBS may undergo cholecystectomy due to symptom overlap (abdominal pain, bloating, altered bowel habits) with biliary disease. Thus, patients with IBS may undergo unnecessary cholecystectomy because their chronic abdominal symptoms are frequently misattributed to gallbladder disease, a phenomenon reflected in the elevated rates of this surgery observed in IBS populations [58,59]. Only two studies (Heaton et al. and Mjåland et al.) assessed and excluded pre-existing IBS preoperatively [22,23]. The remaining studies lacked baseline symptom data, raising misclassification concerns. While MR studies support causality, observational data may partially reflect indication bias. Future studies must include preoperative Rome criteria assessments.

Nevertheless, beyond the mechanistic implications, these findings have important clinical relevance. Cholecystectomy is performed in millions of patients worldwide each year, and our meta-analysis suggests that a substantial proportion may subsequently develop persistent IBS symptoms [60]. Consequently, clinicians should maintain a high index of suspicion for IBS in patients presenting with chronic abdominal pain, bloating, diarrhea, constipation, or altered bowel habits following gallbladder removal, particularly when routine investigations fail to identify an organic cause. Recognition of this potential association is important because IBS can significantly impair quality of life, increase healthcare utilization, and contribute to psychological distress. Furthermore, distinguishing persistent IBS from post-cholecystectomy syndrome or other postoperative complications may facilitate more appropriate management strategies, including dietary interventions, bile acid-directed therapies, pharmacologic treatment, and psychological support when indicated. Increased awareness of the potential long-term gastrointestinal consequences of cholecystectomy may also improve preoperative counseling and help identify patients who could benefit from closer postoperative follow-up.

Several limitations should be considered when interpreting these findings. First, substantial heterogeneity was observed across studies, with high I^2^ values persisting despite subgroup analyses, sensitivity analyses, and meta-regression. This high degree of variability likely stems from multiple interconnected factors that were not consistently reported across studies. The wide range of follow-up durations (0.5 to 12.7 years) may be particularly important, as longer follow-up may allow more time for IBS to develop, while shorter follow-up may miss delayed-onset symptoms. Additionally, the timing of IBS assessment relative to cholecystectomy was inconsistently reported, and it remains unclear whether IBS symptoms represent new-onset conditions or the exacerbation of pre-existing gastrointestinal symptoms. The substantial geographic variation observed, from 2.7% in Norway to 78.8% in Romania, suggests that population-specific factors—including dietary habits, genetic predisposition, healthcare-seeking behavior, and diagnostic practices—may substantially influence prevalence estimates.

Second, considerable variability existed in the diagnostic criteria used for IBS, including the Manning, Rome I, Rome II, Rome III, and Rome IV criteria, and some studies did not clearly report the criteria employed. Differences in IBS definitions, as well as inconsistencies in the timing of IBS assessment following cholecystectomy, may have contributed to the variability in prevalence estimates and effect sizes. Moreover, the various iterations of the Rome criteria exhibit divergent sensitivity and specificity metrics, meaning that diagnostic accuracy varies significantly depending on whether Rome III, Rome IV, or a modified version is employed [61,62]. Additionally, the incomplete reporting of IBS subtypes in several studies (six of 17 studies reported subtype data) limits our ability to determine whether specific subtypes, particularly IBS-D, are preferentially affected by cholecystectomy.

Third, most included studies were observational and therefore susceptible to residual confounding despite adjustment for multiple covariates. Important factors such as diet, psychological comorbidities, medication use, healthcare-seeking behavior, and pre-existing gastrointestinal symptoms may not have been adequately controlled. Furthermore, details regarding the type of cholecystectomy, surgical indications, severity of gallbladder disease, postoperative complications, and IBS subtypes were inconsistently reported, limiting more detailed analyses. While prospective, most cohorts did not assess baseline IBS status using validated criteria, limiting our ability to distinguish new-onset from pre-existing IBS.

Fourth, most of the studies in this meta-analysis reported the crude rather than adjusted effect estimates. Although several included studies reported adjusted analyses, adjustment variables differed substantially between studies and were not consistently available.

Finally, although formal testing did not identify significant publication bias, the relatively small number of studies in some subgroup analyses may have reduced the ability to detect small-study effects. Consequently, the findings should be interpreted with caution and confirmed by large, prospective studies using standardized diagnostic criteria and long-term follow-up.

## 5. Conclusions

This systematic review and meta-analysis, encompassing more than 3.5 million participants across 17 studies, suggests a possible association between cholecystectomy and subsequent IBS. However, no statistically significant difference in the pooled odds of IBS was observed between the cholecystectomy and control groups. The pooled prevalence of IBS among post-cholecystectomy patients was 21%, although this estimate should be interpreted cautiously because substantial between-study heterogeneity (I^2^ ≈ 99%) was observed.

From a clinical perspective, these findings support maintaining awareness of IBS as a potential diagnosis in patients presenting with persistent abdominal pain, bloating, or altered bowel habits after cholecystectomy, particularly after exclusion of structural or organic causes. Future large, prospective studies using standardized diagnostic criteria such as the recently updated Rome V criteria [63] and longer follow-up are needed to better define the relationship between cholecystectomy and IBS, identify patients who may be at greatest risk, and determine whether specific IBS subtypes are preferentially affected.

## Figures and Tables

**Table 1:** Study characteristics.

Study	Country	Study Design	IBS Criteria	Confounders	Follow-Up Time (Years)	IBS Subtypes	Sample Size
**Studies included in the meta-analysis**							
Heaton, 1993 ^†^ [22]	UK	Prospective cohort	Manning	Age	7	NR	85
Mjåland, 2000 [23]	Norway	Prospective cohort	Rome I	Clinical assessment to rule out colonic malignancy	3	NR	487
Mallon, 2006 [24]	UK	Prospective cohort	Rome II	Age at surgery, histological severity	NR	NR	388
Kirk, 2011 ^†^ [25]	UK	Prospective cohort	Manning	Baseline QOL, psychiatric history	2	NR	224
Thistle, 2011 [26]	USA	Prospective cohort	Rome II	Age, sex, GERD, somatization, pain features	1	NR	860
Parkman, 2013 ^†^ [14]	UK	Prospective cohort	Rome III	Age, race, BMI, etiology, comorbidities, medications	NR	NR	391
Ng, 2015 [27]	Australia	Cross-sectional	Rome III	Demographic factors, medical/surgical history	NR	C, D, M, U	396
Amieva-Balmori, 2016 ^†^ [28]	Mexico	Cross-sectional	Rome III	Sociodemographic data	8.5	D	345
Cabrera-Mendoza, 2021 ^†^ [5]	Mexico	Prospective cohort	Rome IV	Age- and sex-matching	1	C, D, M	332
Georgescu, 2022 [29]	Romania	Cross-sectional	Rome III	Genetic variants; GWAS adjusted for age, sex, population stratification	1	NR	104
Chang, 2023 ^†^ [30]	Republic of Korea	Prospective cohort	Rome III	Age, residence, BMI, comorbidities (DM, NAFLD), diet	1	NR	261
Zhao, 2023 ^†^ [32]	China	Prospective cohort	Rome III	Sociodemographics, health behaviors, comorbidities, medications	12.7	D	413,472
Black, 2025 [33]	Multinational	Cross-sectional	Rome IV	Demographics, prior medical diagnoses, psychological symptoms	NR	C, D, M, U	2195
**Studies included in the systematic review only**							
Hearing, 1999 [34]	UK	Prospective cohort	Manning	Fiber intake, use of constipating drugs, non-specific effects of surgery	0.5	NR	106
Chen, 2023 [31]	China	MR	Not stated	Sex, age, BMI, SSC score, coping strategies	NR	NR	462,933
Peng, 2024 [13]	China	MR	Not stated	Genetic variants; original GWAS adjusted for demographics	NR	D	949,611
Niu, 2025 [7]	China	MR	Rome II	Genetic variants; principles of MR ensure independence from confounders	NR	C, D	108

^†^ Studies included in both the meta-analysis on the odds of IBS and cholecystectomy and the meta-analysis on the prevalence of IBS in patients with cholecystectomy. IBS—irritable bowel syndrome; QOL—quality of life; GERD—gastroesophageal reflux disease; BMI—body mass index; GWAS—genome-wide association study; DM—diabetes mellitus; NAFLD—non-alcoholic fatty liver disease; SSC—somatization symptom checklist; MR—Mendelian randomization; NR—not reported; C—constipation-predominant; D—diarrhea-predominant; M—mixed; U—unclassified.

## Data Availability

All data used in this analysis can be found in the following databases: PubMed, Embase, Scopus, and Web of Science.

## Supplementary Materials

The following supporting information can be downloaded at: https://www.mdpi.com/article/10.3390/jcm15145392/s1, PRISMA 2020 Checklist.

## Abbreviations

The following abbreviations are used in this manuscript:

IBS	Irritable bowel syndrome
DGBI	Disorder of gut–brain interaction
PRISMA	Preferred Reporting Items for Systematic Reviews and Meta-Analyses
PICO	Population, Intervention, Comparison, Outcome
ORs	Odds ratios
CI	Confidence intervals
NOS	Newcastle–Ottawa Scale
GERD	Gastroesophageal reflux disease
BMI	Body mass index
GWAS	Genome-wide association study
QOL	Quality of life
DM	Diabetes mellitus
NAFLD	Non-alcoholic fatty liver disease
SSC	Somatization symptom checklist
BAM	bile acid malabsorption
SIBO	Small intestinal bacterial overgrowth
MR	Mendelian randomization
Q-Genie	Quality of Genetic Association Studies
